# Cost-effectiveness analysis of dostarlimab plus carboplatin-paclitaxel as first-line treatment for advanced endometrial cancer

**DOI:** 10.3389/fimmu.2023.1267322

**Published:** 2023-09-04

**Authors:** Maojin You, Xiaoling Zeng, Jinrong Zhang, Yufan Huang, Yalan Zhang, Zhongjie Cai, Yingying Hu

**Affiliations:** ^1^ Department of Pharmacy, Mindong Hospital Affiliated to Fujian Medical University, Ningde, Fujian, China; ^2^ Department of Pharmacy, The Second Hospital of Zhangzhou, Zhangzhou, Fujian, China; ^3^ Department of Pharmacy, The Second Affiliated Hospital of Fujian Medical University, Quanzhou, Fujian, China; ^4^ Department of Pharmacy, Mengchao Hepatobiliary Hospital of Fujian Medical University, Fuzhou, Fujian, China

**Keywords:** dostarlimab, carboplatin-paclitaxel, cost-effectiveness, first-line treatment, endometrial cancer

## Abstract

**Background:**

A recent phase III clinical trial (NCT03981796) evaluated the efficacy and safety of dostarlimab combined with carboplatin-paclitaxel (DOS-CP) compared to placebo combined with carboplatin-paclitaxel (PLB-CP) as a first-line treatment for advanced endometrial cancer (EC). The NCT03981796 trial demonstrated that DOS-CP significantly improved progression-free survival and overall survival of patients with advanced EC while maintaining an acceptable safety profile. However, DOS-CP is expensive and its cost-effectiveness has not been evaluated. This study aims to evaluate the cost-effectiveness of DOS-CP compared to PLB-CP as a first-line treatment for advanced EC from the perspective of the Chinese healthcare system.

**Methods:**

A Markov model with three health states was developed to evaluate the cost-effectiveness of DOS-CP as a first-line treatment for advanced EC. Clinical efficacy data were derived from the NCT03981796 trial, and drug costs were determined based on national tender prices. Other costs and utility values were obtained from published literature. The outcomes assessed included total costs, quality-adjusted life years (QALYs), and incremental cost-effectiveness ratios (ICERs). The robustness of the model was assessed through one-way sensitivity analysis and probabilistic sensitivity analysis.

**Results:**

In comparison to PLB-CP, the ICER of DOS-CP was $98,276.61/QALY for the overall population, $53,063.61/QALY for the dMMR subgroup, and $124,088.56/QALY for the pMMR subgroup. All of these ICER values were higher than the willingness-to-pay threshold of $38,201 per QALY. The most important variable that affected the results of the model was the discount rate, the cost of dostarlimab, and the utility value for progressive disease.

**Conclusion:**

From the perspective of the Chinese healthcare system, DOS-CP is unlikely to be a cost-effective first-line treatment option for advanced EC.

## 1 Introduction

Endometrial cancer (EC) ranks as the sixth most prevalent malignant tumor among women globally and is the second most common gynecological cancer, second only to cervical cancer (1). China is also a high-incidence region for EC, with nearly 84,520 new cases reported in 2022 (2). In recent years, there has been a growing concern regarding the trend of EC affecting younger age groups and its increasing incidence (3, 4). It is also worrying that about 10% to 15% of patients with EC are determined to be at an advanced stage at the time of initial diagnosis, and their 5-year survival rate is only 10% to 20% (5).

The standard first-line treatment regimen for advanced EC patients is carboplatin plus paclitaxel (6). However, these patients have a poor prognosis, with a median overall survival (OS) of less than 3 years (7). Therefore, the search for new treatment options is of paramount importance. In recent years, immunotherapy has emerged as an attractive therapeutic choice and has shown promising efficacy (8, 9). Additionally, cytotoxic chemotherapy can exert immunomodulatory effects, such as disrupting immune inhibitory pathways and enhancing cytotoxic T-cell responses. Thus, the combination of chemotherapy and immunotherapy may have a synergistic effect within the tumor microenvironment (10–12). Dostarlimab, an immune checkpoint inhibitor (ICI), blocks the interaction between programmed death-ligand 1 (PD-L1) and programmed death 1 (PD-1), thereby restoring the immune cells’ ability to attack cancer cells (13). Mirza et al. (14) conducted a phase III clinical trial (NCT03981796) to evaluate the efficacy and safety of dostarlimab in combination with carboplatin-paclitaxel (DOS-CP) compared to placebo plus carboplatin-paclitaxel (PLB-CP) in patients with primary advanced or recurrent EC. The results showed that DOS-CP significantly increased the progression-free survival (PFS) and OS in the overall population, as well as in the mismatch repair-deficient/microsatellite instability-high (dMMR-MSI-H) and the mismatch repair-proficient/microsatellite-stable (pMMR-MSS) subgroup of patients with primary advanced or recurrent EC. Moreover, the adverse reactions were manageable.

Although DOS-CP has a great survival advantage over PLB-CP for advanced EC, the DOS-CP regimen leads to an increase in healthcare costs for society, which is a non-negligible problem for countries with limited healthcare resources such as China. Therefore, it is necessary to evaluate the cost-effectiveness of DOS-CP for the treatment of advanced EC to assess its affordability to society and accessibility to patients. This study aimed to evaluate the economics of DOS-CP as a first-line treatment option for advanced EC compared to PLB-CP from the perspective of the Chinese healthcare system based on the NCT03981796 trial (14). We have provided the following articles in response to the Comprehensive Health Economic Evaluation Reporting Standards (CHEERS) 2022 Report List (15).

## 2 Methods

### 2.1 Modeling

We have established a Markov model by TreeAge Pro 2022 (TreeAge Software, LLC, USA) to assess the cost and effectiveness of two treatment options, DOS-CP and PLB-CP, as first-line therapies for patients with advanced EC. The survival data used in the model are derived from the NCT03981796 trial (14). The study population includes the overall population, dMMR–MSI-H subgroup, and pMMR–MSS subgroup. Due to the unavailability of individual patient data, we utilized GetData Graph Digitizer software (version 1.2) to digitize the Kaplan-Meier curves from the NCT03981796 trial (14). Subsequently, following the methodology described by Hoyle et al. (16), we used the “survival,” “survHE,” and “survminer” packages in R software to fit and infer the survival function beyond the follow-up time using the following survival distributions: exponential, Weibull, log-logistic, and log-normal distributions (17, 18). The identification of the most suitable survival distribution was guided by the Akaike information criterion (AIC) and Bayesian information criterion (BIC), whereby a superior fit is characterized by lower AIC and BIC values (19, 20). The AIC and BIC values for different survival distributions of the PFS and OS curves are provided in **Supplementary Table B**. The selected survival distribution and corresponding data are presented in **Table 1**. The probability of patients reaching death in the context of PFS was assumed to be the background mortality rate in China for the year 2022 (21).

**Table 1:** Relevant parameters of survival distribution.

Variable	Value	Source
PFS model for the overall population		
DOS-CP group	log-logistic: Scale= 0.07141, Shape=1.44712	(14)
PLB-CP group	log-logistic: Scale=0.11100, Shape=1.97022	(14)
OS model for the overall population		
DOS-CP group	log-logistic: Scale= 0.02016, Shape= 1.25053	(14)
PLB-CP group	log-logistic: Scale=0.03480, Shape= 1.66018	(14)
PFS model for dMMR-MSI-H subgroup		
DOS-CP group	log-logistic: Scale= 0.02866, Shape=0.87189	(14)
PLB-CP group	log-logistic: Scale=0.12359, Shape=2.07275	(14)
OS model for dMMR-MSI-H subgroup		
DOS-CP group	Weibull: Scale=0.00409, Shape= 0.78301	(14)
PLB-CP group	log-logistic: Scale=0.03036, Shape= 1.38327	(14)
PFS model for pMMR-MSS subgroup		
DOS-CP group	log-logistic: Scale= 0.08387, Shape=1.62799	(14)
PLB-CP group	log-logistic: Scale=0.10865 Shape=1.89199	(14)
OS model for pMMR-MSS subgroup		
DOS-CP group	Weibull: Scale=0.02526, Shape= 1.40300	(14)
PLB-CP group	log-logistic: Scale=0.03641, Shape= 1.80437	14)

dMMR-MSI-H, mismatch repair-deficient/microsatellite instability-high; DOS-CP, dostarlimab plus carboplatin-paclitaxel; PD, progressive disease; PFS, progression-free survival; PLB-CP, placebo plus carboplatin-paclitaxel; pMMR-MSS, mismatch repair-proficient/microsatellite-stable; OS, overall survival.

**Figure 1:**
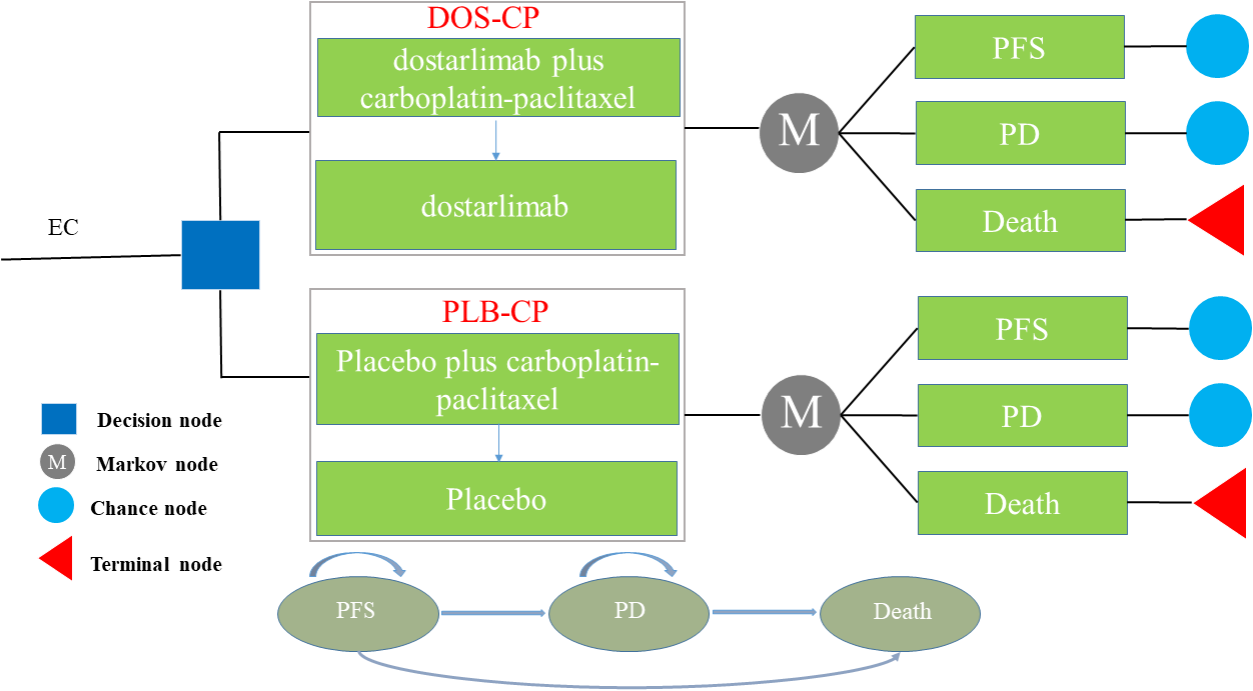
The Markov model simulating outcomes for the NCT03981796 trial. All patients started with PFS state and received treatment with DOS-CP or PLB-CP. DOS-CP, dostarlimabplus carboplatin-paclitaxel; EC, endometrial cancer; PD, progressive disease; PFS, progression-free survival; PLB-CP, placebo plus carboplatin-paclitaxel.

The Markov model comprises three distinct health states, namely PFS, PD, and death (**Figure 1**). We assume that all patients enter the model with PFS as their initial state (22). Once the model is initiated, patients either remain in their current health state or transition to a new state, with no possibility of returning to previous health states. The model runs for approximately 20 years, with both treatment groups experiencing a mortality rate exceeding 95% at that time point. Each cycle in the model has a fixed duration of 21 days. The outcomes of the model include total costs, quality-adjusted life years (QALYs), and incremental cost-effectiveness ratios (ICERs). Following the recommendations of the Guidelines for Pharmacoeconomic Evaluations in China (23), we have chosen three times the per capita GDP of China in 2022 as the threshold for willingness-to-pay (WTP), equivalent to $38,201/QALY. If the ICER of a treatment strategy is lower than the predetermined WTP threshold, it is considered cost-effective.

### 2.2 Clinical data

We extracted data on clinical efficacy and adverse reactions from the NCT03981796 trial (14). The enrolled patients met the following criteria: 1) age ≥18; 2) histologically or cytologically confirmed primary advanced or recurrent EC that is not amenable to curative treatment. After enrollment, patients were randomized in a 1:1 ratio to receive either dostarlimab (500mg) or placebo combined with carboplatin (area under the curve of 5mg/ml/minute) and paclitaxel (175mg/m2 body surface area) (DOS-CP or PLB-CP group) treatment. The treatment was administered intravenously every 3 weeks for the first 6 cycles, followed by intravenous dostarlimab (1000mg) or placebo every 6 weeks for a duration of 3 years or until disease progression. According to the results of the NCT03981796 trial, the median duration of treatment for patients in the DOS-CP and PLB-CP groups was 43 weeks and 36 weeks, respectively. Since detailed treatment data for patients experiencing disease progression were not provided in the NCT03981796 trial, we assumed that all patients received the best supportive care (BSC) after disease progression.

### 2.3 Cost and utility

In our study, we have considered solely direct medical costs, which encompass expenses related to drugs, tests, routine follow-up, terminal care during end-of-life, BSC, and the management of grade 3 or higher adverse events with an incidence greater than 5% **Table 2**). As dostarlimab is not yet available on the market in China, we used the price of pembrolizumab in China, a PD-1 inhibitor approved by the FDA for the treatment of advanced EC, as a reference for the cost estimation. The costs of other drugs were sourced from national tender prices, and other expenses were obtained from published literature and adjusted to 2022 values using the Medical Price Index from the National Bureau of Statistics in China (21). All costs were presented in US dollars and converted at the average exchange rate of $1 to 6.73 CNY in 2022. Since the NCT03981796 trial did not provide relevant data on quality of life, utility values for PFS and PD in this study were obtained from published literature in China. We also considered the disutility of grade 3 or higher adverse events with an incidence greater than 5% (**Table 2**). Discounting was applied to both costs and utilities in this study, with a discount rate of 5% (23).

**Table 2:** Basic parameters of the input model and the range of sensitivity analyses.

Variable	Base Value	Range		Distribution	Source
		Min	Max		
DOS-CP group: Incidence of AEs					
Anemia	14.94%	11.9%	17.93%	Beta	(14)
Neutropenia	9.54%	7.63%	11.45%	Beta	(14)
Neutrophil count decreased	8.30%	6.64%	9.96%	Beta	(14)
White-cell count decreased	6.64%	5.31%	7.97%	Beta	(14)
PLB-CP group: Incidence of AEs					
Anemia	14.63%	11.7%	17.56%	Beta	(14)
Neutropenia	9.35%	7.48%	11.22%	Beta	(14)
Neutrophil count decreased	8.13%	6.50%	9.76%	Beta	(14)
White-cell count decreased	6.50%	5.20%	7.80%	Beta	(14)
Cost, $					
Carboplatin (100mg)	24.96	19.97	29.95	Gamma	(24)
Paclitaxel (100mg)	102.53	82.02	123.04	Gamma	(24)
Pembrolizumab (100mg)	2662.41	2129.93	3194.89	Gamma	(24)
Dostarlimab (500mg)	5324.82	4259.86	6389.78	Gamma	(24)
Best supportive care per cycle	182.23	145.78	218.68	Gamma	(25)
Routine follow-up per cycle	73.72	58.98	88.46	Gamma	(25)
Test per cycle	357.34	285.87	428.81	Gamma	(26)
Terminal care in end-of-life	1489.51	1191.61	1787.41	Gamma	(18)
Neutropenia	454.26	363.41	545.11	Gamma	(26)
Anemia	336.63	269.30	403.96	Gamma	(27)
Neutrophil count decreased	454.26	363.41	545.11	Gamma	(26)
White-cell count decreased	210.85	168.68	253.02	Gamma	(22)
Utility value					
PFS	0.817	0.654	0.980	Beta	(28)
PD	0.779	0.623	0.935	Beta	(28)
Disutility due to AEs					
Neutropenia	0.2	0.160	0.240	Beta	(29)
Anaemia	0.07	0.056	0.084	Beta	(30)
Neutrophil count decreased	0.2	0.160	0.240	Beta	(29)
White-cell count decreased	0.066	0.053	0.079	Beta	(31)
Body surface area (m^2^)	1.69	1.35	2.03	Normal	(32)
Weight (Kg)	59.00	47.20	70.80	Normal	(33)
Discount rate	0.05	0.00	0.08	Fixed	(23)
Creatinine clearance rate (ml/min)	70	56	84	Gamma	(34)

AE, adverse event; DOS-CP, dostarlimab plus carboplatin-paclitaxel; PD, progressive disease; PFS, progression-free survival; PLB-CP, placebo plus carboplatin-paclitaxel; OS, overall survival.

### 2.4 Sensitivity analysis

In this study, one-way sensitivity analysis and probabilistic sensitivity analysis were conducted to assess the robustness of the model. In the one-way sensitivity analysis, variables were adjusted within the reported ranges from the literature, and in the absence of data, a ±20% variation from the base value was applied. The discount rate varied from 0% to 8% (**Table 2**). The results of the one-way sensitivity analysis were presented using tornado diagrams. To assess the impact of variables uncertainty on the model results, we performed 1000 iterations of Monte Carlo simulation, with parameters sampled from their specified distributions **Table 2**). The results of the probabilistic sensitivity analysis were presented using the Cost-effectiveness acceptability curve and scatter plots. In addition, we made DOS-CP cost-effective compared to PLB-CP by gradually reducing the price of dostarlimab to obtain the dostarlimab cost threshold.

### 2.5 Scenario analysis

We conducted three scenario analyses in the overall population. Scenario 1 involved varying the model duration to 3 years, 5 years, 10 years, and 15 years to evaluate the impact of model duration on the results. In Scenario 2, we assumed that only 80% or 50% of patients received BSC after disease progression, aiming to simulate treatment discontinuation for some patients in clinical practice due to various reasons. Scenario 3 involved reducing the price of dostarlimab from the originally assumed price to 80%, 50%, and 20% to assess the influence of different dostarlimab prices on the cost-effectiveness of DOS-CP.

## 3 Results

### 3.1 Base case analysis

The results are shown in **Table 3**. In the overall population, compared to the PLB-CP, the DOS-CP had an incremental effect of 1.49 QALYs and an incremental cost of $146,182.58, resulting in an ICER of $98,276.61 per QALY. In the dMMR-MSI-H and pMMR-MSS subgroups, compared to the PLB-CP, the DOS-CP had incremental costs of $220,465.51 and $128,081.44, incremental effects of 4.16 QALYs and 1.03 QALYs, resulting in ICERs of $53,063.61/QALY and $124,088.56/QALY, respectively. In China, with a WTP threshold of $38,201 per QALY, DOS-CP as a first-line treatment for advanced EC is not cost-effective compared to PLB-CP in the overall population, dMMR-MSI-H subgroup, and pMMR-MSS subgroup.

**Table 3:** The cost and outcome results of the base-case analysis.

Regimen	overall population		dMMR-MSI-H subgroup		pMMR-MSS subgroup	
	DOS-CP	PLB-CP	DOS-CP	PLB-CP	DOS-CP	PLB-CP
Total cost ($)	181227.07	35044.49	262527.61	42062.1	160589.85	32508.41
Incremental costs ($)	146182.58	–	220465.51	–	128081.44	–
Total effectiveness (QALYs)	4.02	2.54	7.17	3.01	3.39	2.36
Incremental effectiveness (QALYs)	1.49	–	4.16	–	1.03	–
ICER ($/QALY)	198276.61	–	53063.61	–	124088.56	–

dMMR-MSI-H, mismatch repair-deficient/microsatellite instability-high; DOS-CP, dostarlimab plus carboplatin-paclitaxel; ICER, increase cost-effectiveness ratio; PLB-CP, placebo plus carboplatin-paclitaxel; pMMR-MSS, mismatch repair-proficient/microsatellite-stable; QALY, quality-adjusted life year.

### 3.2 Sensitivity analysis

**Figure 2:**
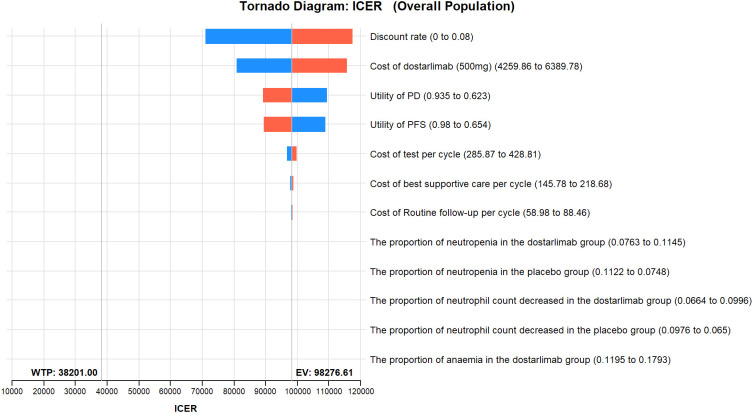
One-way sensitivity analyses of DOS-CP in comparison to PLB-CP in the overall population. DOS-CP, dostarlimabplus carboplatin-paclitaxel; ICER, incremental cost-effectiveness ratio; PD, progressive disease; PFS, progression-free survival; PLB-CP, placebo plus carboplatin-paclitaxel.

**Figure 3:**
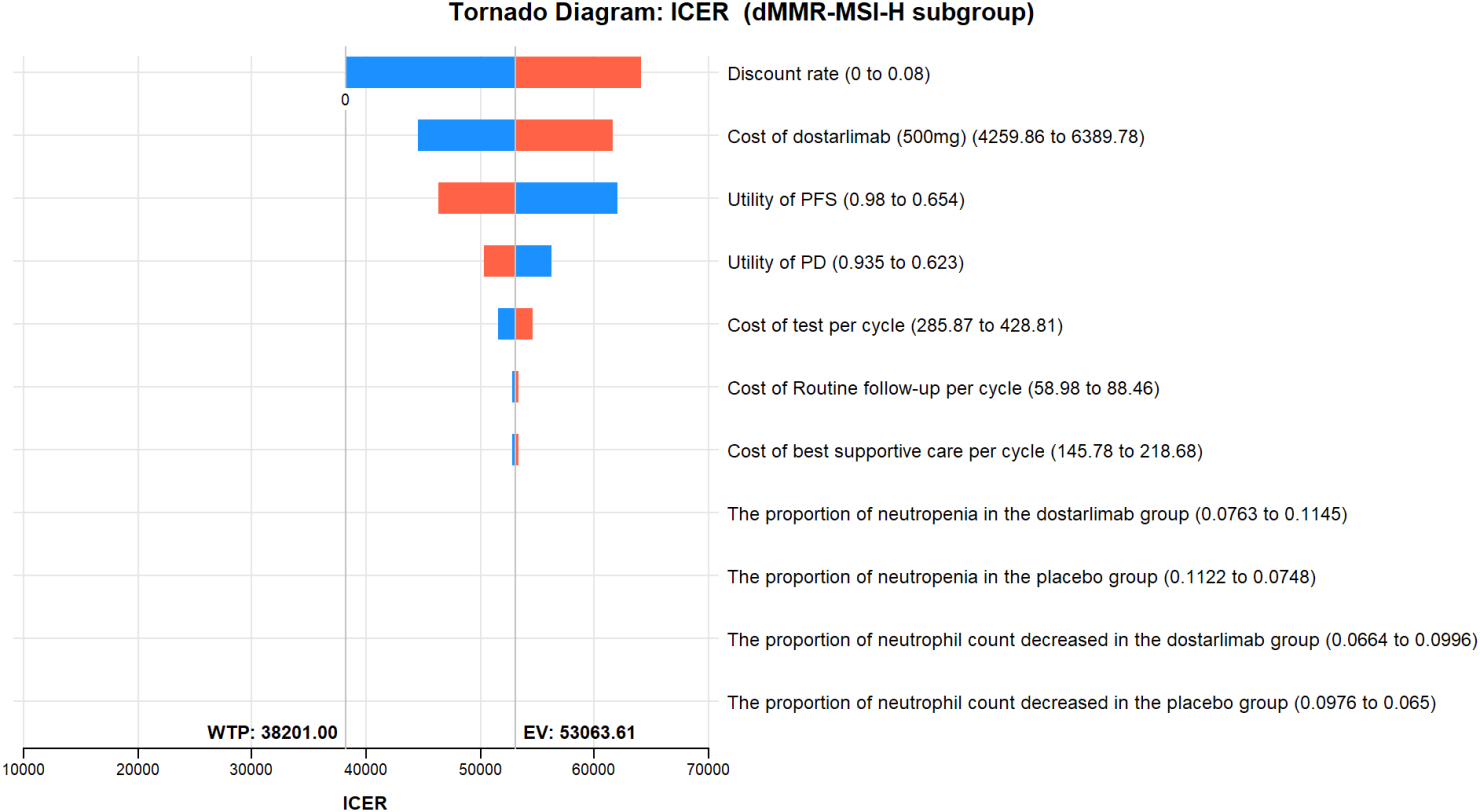
One-way sensitivity analyses of DOS-CP in comparison to PLB-CP in dMMR-MSI-H subgroup. DOS-CP, dostarlimabplus carboplatin-paclitaxel; ICER, incremental cost-effectiveness ratio; PD, progressive disease; PFS, progression-free survival; PLB-CP, placebo plus carboplatin-paclitaxel.

**Figure 4:**
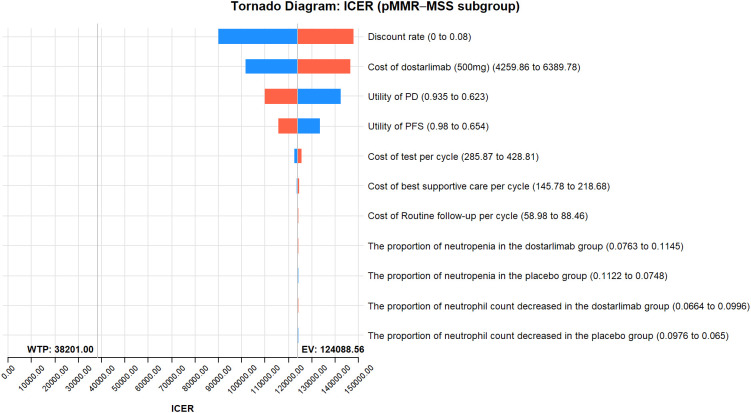
One-way sensitivity analyses of DOS-CP in comparison to PLB-CP in the pMMR-MSS subgroup. DOS-CP, dostarlimabplus carboplatin-paclitaxel; ICER, incremental cost-effectiveness ratio; PD, progressive disease; PFS, progression-free survival; PLB-CP, placebo plus carboplatin-paclitaxel.

**Figure 5:**
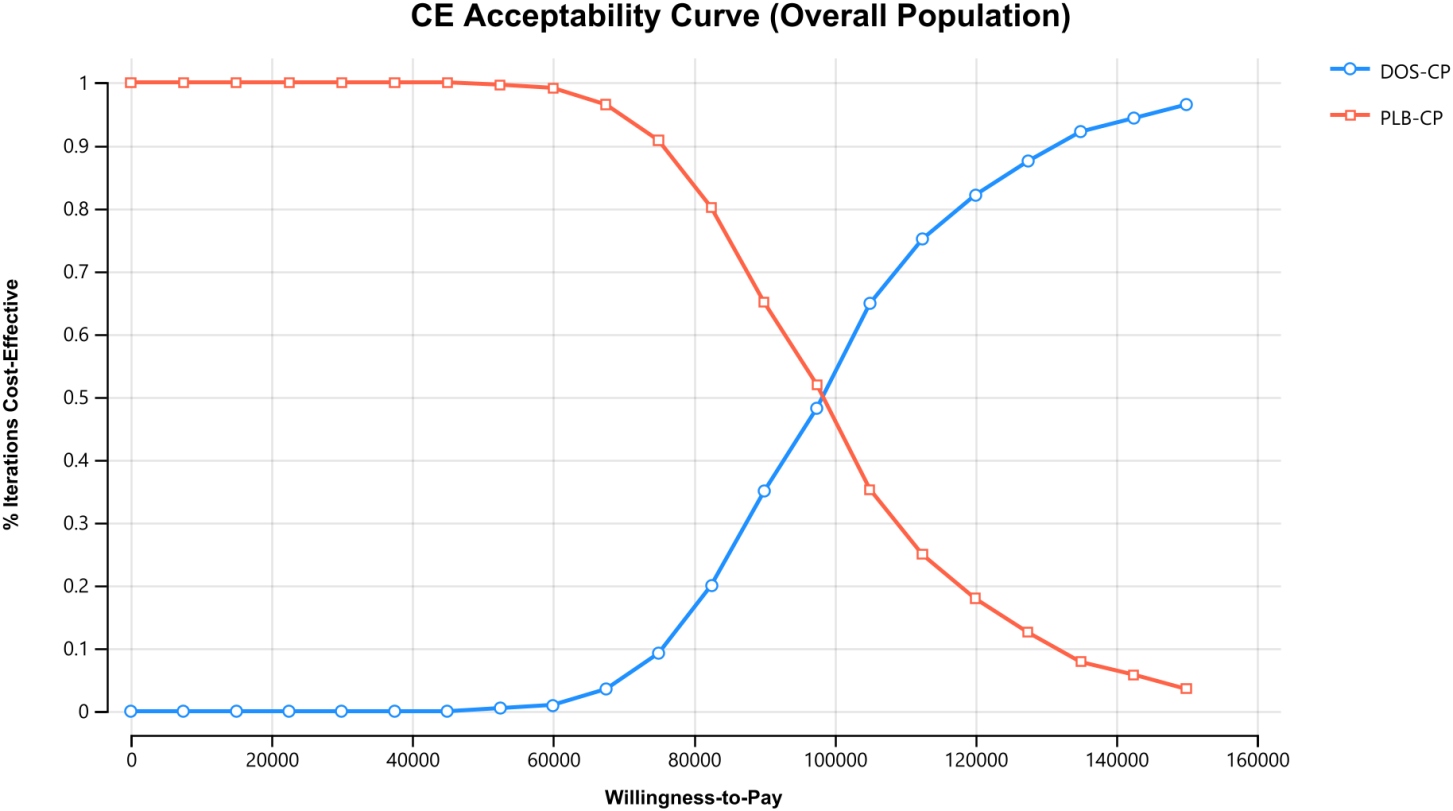
The cost-effectiveness acceptability curves in the overall population. DOS-CP, dostarlimabplus carboplatin-paclitaxel; PLB-CP, placebo plus carboplatin-paclitaxel.

**Figure 6:**
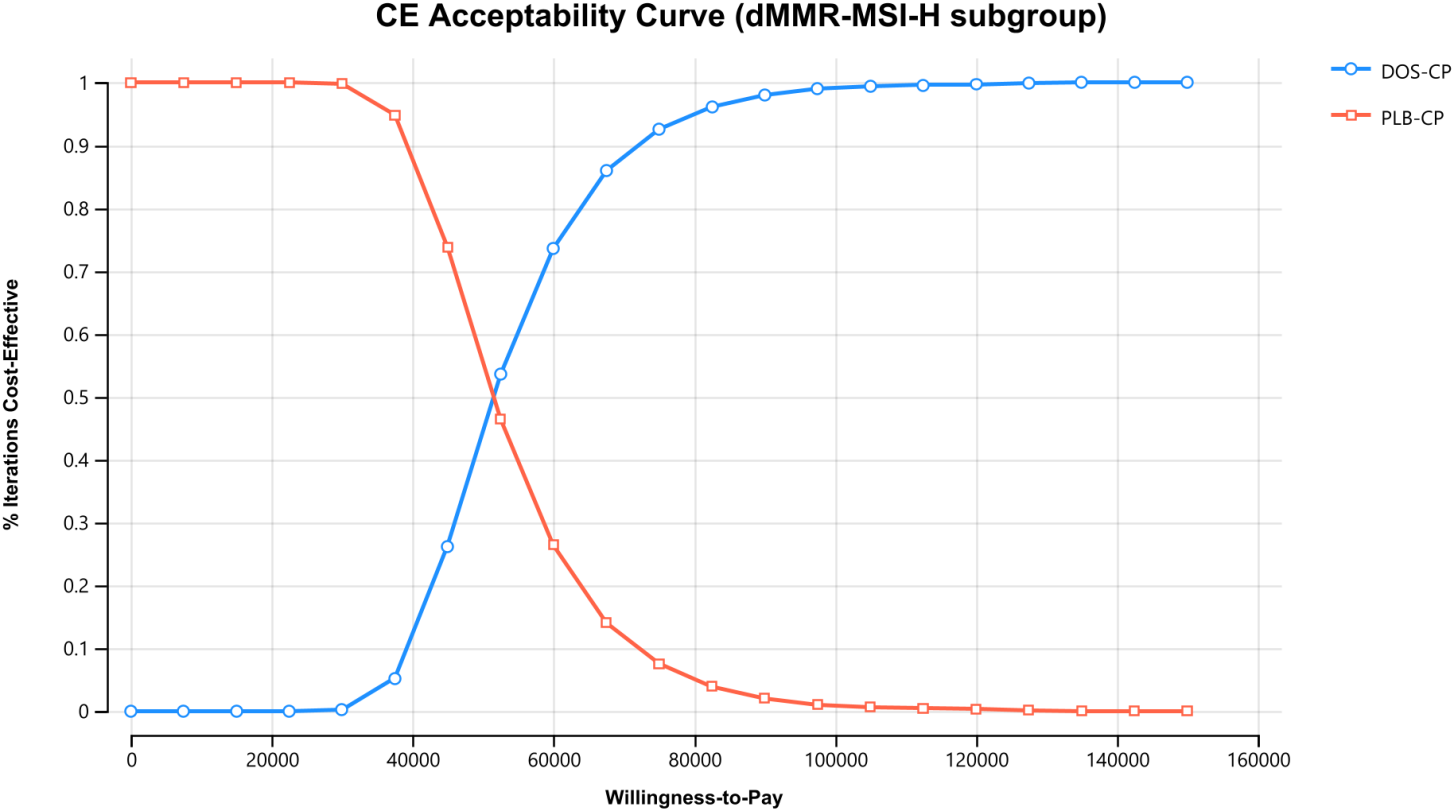
The cost-effectiveness acceptability curves in the dMMR-MSI-H subgroup. DOS-CP, dostarlimabplus carboplatin-paclitaxel; PLB-CP, placebo plus carboplatin-paclitaxel.

**Figure 7:**
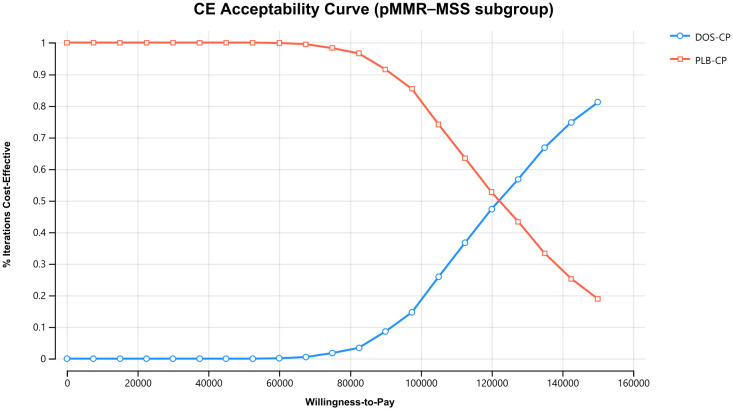
The cost-effectiveness acceptability curves in the pMMR-MSS subgroup. DOS-CP, dostarlimabplus carboplatin-paclitaxel; PLB-CP, placebo plus carboplatin-paclitaxel.

The results of the one-way sensitivity analysis are presented in the tornado diagram (**Figures 2**–**4**). The most influential variables are the discount rate, the cost of dostarlimab, and the utility value of PD. However, The ICER is always higher than our pre-determined WTP threshold when these variables vary within a given range, indicating that such variations do not affect the model results. The remaining variables have a relatively minor impact on the model. The findings from the probabilistic sensitivity analysis are represented in **Figures 5**–**7**, as well as **Supplementary Figures B-D**. At a WTP threshold of $38,201 per QALY, the probability of DOS-CP being cost-effective compared to PLB-CP is 6.9% in the dMMR-MSI-H subgroup, while it is 0% in the overall population and the pMMR-MSS subgroup. Furthermore, in the dMMR-MSI-H subgroup, DOS-CP becomes a cost-effective first-line treatment for advanced EC compared to PLB-CP when the price of dostarlimab falls below $3,468.5, while in the overall population and pMMR-MSS subgroup, the price of dostarlimab needs to drop below $1,639.4 and $1,241.3, respectively, for DOS-CP to be considered a cost-effective treatment option for advanced EC.

### 3.3 Scenario analysis

In Scenario 1, when the modeled durations are varied to 3, 5, 10, and 15 years, the ICERs for DOS-CP are $619452.15/QALY, $274,562.10/QALY, $13,8002.65/QALY, and $109,491.00/QALY, respectively, compared to PLB-CP. In Scenario 2, when disease progression occurred and only 80% or 50% of patients received BSC, the ICER of DOS-CP compared to PLB-CP was $97,862.65/QALY or $97,241.72/QALY, respectively. In Scenario 3, when the price of dostarlimab decreased to 80%, 50%, or 20% of the original price, the ICER of DOS-CP compared to PLB-CP was $80,916.91/QALY, $54,877.36/QALY, and $28,837.81/QALY, respectively (**Table 4**).

**Table 4:** Scenario analyses in overall population.

Scenarios	Cost ($)		QALY		ICER ($/QALY)
	DOS-CP group	PLB-CP group	DOS-CP group	PLB-CP group	
Scenario 1					
Model runtime (year) =3	151887.04	22324.40	1.78	1.57	619452.15
Model runtime (year) =5	161109.36	27499.30	2.46	1.97	274562.10
Model runtime (year) =10	172676.94	32544.81	3.36	2.35	138002.65
Model runtime (year) =15	178227.32	34275.09	3.79	2.48	109491.00
Scenario 2					
80% patients receive BSC	179275.47	33708.64	4.02	2.54	97862.65
50% patients receive BSC	176348.09	31704.86	4.02	2.54	97241.72
Scenario 3					
80% dostarlimab price	155405.20	35044.49	4.02	2.54	80916.91
50% dostarlimab price	116672.40	35044.49	4.02	2.54	54877.36
20% dostarlimab price	77939.60	35044.49	4.02	2.54	28837.81

BSC, best supportive care; DOS-CP, dostarlimabplus carboplatin-paclitaxel; ICER, increase cost-effectiveness ratio; PLB-CP, placebo plus carboplatin-paclitaxel; QALY, quality-adjusted life year.

## 4 Discussion

Despite advances in the multidisciplinary treatment of EC, the options for advanced EC remain limited and the prognosis is poor (35). The NCT03981796 trial compared the efficacy of DOS-CP and PLB-CP as first-line treatment for advanced EC, and the results showed that the DOS-CP group had significantly longer PFS and OS than the PLB-CP group in the overall population, dMMR-MSI-H subgroup, and pMMR-MSS subgroup. This finding addresses the need for effective treatment in advanced EC patients. However, an important concern for healthcare decision-makers, physicians, and patients is that new alternative treatment options, such as immunotherapy and molecularly targeted therapies, often come at a higher cost than previously used therapies, leading to sustained increases in healthcare costs (36, 37). The price of new anticancer drugs should not only be reasonable and affordable for patients to easily access treatment but also sustainable for the national healthcare system and pharmaceutical companies. Therefore, evaluating the cost-effectiveness of DOS-CP for the treatment of advanced EC is essential.

Our study findings revealed that the ICER of DOS-CP compared to PLB-CP was higher than the pre-defined WTP threshold in the overall population, dMMR-MSI-H subgroup, and pMMR-MSS subgroup. Sensitivity analyses demonstrated the robustness of the model results. Therefore, for patients with advanced EC in China, DOS-CP is unlikely to be a cost-effective first-line treatment option compared to PLB-CP. However, these research findings should not be considered as a basis to restrict the utilization of dostarlimab, as it may lead to missed opportunities for beneficial treatment options. Instead, they should be regarded as economic considerations for informing the implementation of China’s national pricing negotiation policies (38, 39). China has initiated a policy of banded purchasing of drugs to reduce drug costs. Such a policy may lead to a significant increase in the probability of cost-effectiveness of DOS-CP. In addition, the NCT03981796 trial showed that treatment with DOS-CP reduced the risk of progression or death by 72% in the dMMR-MSI-H subgroup and by 36% in the overall population, whereas the pMMR-MSS subgroup showed even less of a survival benefit, compared with PLB-CP. This may be explained by the increased expression of the PD-1 receptor and its ligands (PD-L1 and PD-L2) and high tumor mutational burden in the dMMR-MSI-H subtype, making them potentially sensitive to treatment with PD-1 inhibitors and PD-L1 inhibitors. Consistent with this, the results of our cost-effectiveness analysis showed that the dMMR-MSI-H subgroup had a lower ICER than the whole population, whereas the pMMR-MSS subgroup had a higher ICER than both the whole population and the dMMR-MSI-H subgroup. Therefore, it is important to detect MMR and MSI status, and biomarkers that accurately predict the best response may also be another way to improve the cost-effectiveness of the DOS-CP strategy for advanced EC. Such findings will provide an important reference for China’s health insurance policymakers to price dostarlimab and approve appropriate indications after its launch.

To the best of our knowledge, there is only one cost-effectiveness analysis for the use of novel anticancer drugs for EC in China (28). The results of this study indicated that, from the perspective of the Chinese healthcare system, lenvatinib plus pembrolizumab for the treatment of advanced EC patients with pMMR who experienced disease progression after receiving platinum-based chemotherapy is not considered cost-effective, compared to chemotherapy. There was also only one study on the cost-effectiveness of dostarlimab (40), the results of which indicated that dostarlimab was not cost-effective compared with chemotherapy in patients with recurrent dMMR EC in the United States based on a willingness-to-pay threshold of $100,000/QALY. These results are consistent with our findings.

The strengths of our study deserve highlighting. Firstly, to the best of our knowledge, this is the first cost-effectiveness analysis evaluating the use of dostarlimab as a first-line treatment for advanced EC using a Markov model, and our findings have implications not only for China but also for other countries. Secondly, we conducted subgroup analyses for patients with different MMR and MSI statuses in advanced EC, including the dMMR-MSI-H and the pMMR-MSS subgroup, to provide insights into the cost-effectiveness of dostarlimab in specific patient populations. Additionally, we performed scenario analyses in the overall population that captured a range of clinical practice scenarios, thereby enhancing the applicability and generalizability of our findings. However, our study also has certain limitations that should be acknowledged. Firstly, there are limitations in the data sources as we were unable to obtain long-term survival data beyond the follow-up period of clinical trials. We utilized a survival model to simulate data beyond the follow-up period, which may introduce biases compared to actual data. Our cost-effectiveness analysis will be updated once long-term survival data becomes available. Secondly, in the absence of second-line treatment data, we assumed that all patients would receive BSC after disease progression. This may not accurately reflect the real clinical scenario. Thirdly, our model only included serious adverse events of Grade 3 or above with an incidence rate greater than 5%. However, sensitivity analyses showed that changes in the probability of serious adverse events would not significantly impact our results. Lastly, the NCT03981796 trial did not provide data on health-related quality of life, and the utility values used in our study were derived from published literature in China, which may introduce biases in the model results. Despite these limitations, our study findings still provide valuable economic insights for decision-makers and serve as evidence for drug pricing aftermarket launch.

## 5 Conclusion

From the perspective of the Chinese healthcare system, DOS-CP is not cost-effective compared to PLB-CP as a first-line treatment strategy for advanced EC in the overall population, dMMR-MSI-H subgroup, and pMMR-MSS subgroup. At a WTP threshold of $38,201 per QALY, in the overall population, dMMR-MSI-H subgroup, and pMMR-MSS subgroup, the price of dostarlimab (500 mg) would need to fall below $1,639.4, $3,468.5, and $1,241.3, respectively, for DOS-CP to become a cost-effective first-line treatment option for advanced EC, compared to PLB-CP.

## Data availability statement

The original contributions presented in the study are included in the article/**Supplementary Material**. Further inquiries can be directed to the corresponding authors.

## Author contributions

MY: Writing – original draft. XZ: Writing – review & editing. JZ: Writing – review & editing. YFH: Writing – review & editing. YZ: Writing – review & editing. ZC: Writing – review & editing. YYH: Writing – review & editing.
