# Supplementary material for: Cost-effectiveness analysis of dostarlimab plus carboplatin-paclitaxel as first-line treatment for advanced endometrial cancer
Source: Front Immunol. 2023 Sep 4;14:1267322. doi: 10.3389/fimmu.2023.1267322 (PMC10507332; doi:10.3389/fimmu.2023.1267322)
Supplement: Supplementary file 1 [file DataSheet_1.docx]

Supplementary Material

**Cost-effectiveness analysis of dostarlimab plus carboplatin-paclitaxel as first-line treatment for advanced endometrial cancer**

**1. Supplementary Table 1. CHEERS 2022 Checklist.**

**2. Supplementary Table 2. Comparison of survival models.**

**3. Supplementary Figure 1. Results of the survival curve fit the DOS-CP group and PLB-CP group.**

**4. Supplementary Figure 2: Probabilistic sensitivity analysis for the overall population.**

**5. Supplementary Figure 3: Probabilistic sensitivity analysis for the dMMR-MSI-H subgroup.**

**6. Supplementary Figure 4: Probabilistic sensitivity analysis for the pMMR-MSS subgroup.**

1. **Supplementary Table A. CHEERS 2022 Checklist.**

| **Topic** | **No.** | **Item** | **Reported** |
| --- | --- | --- | --- |
| **Title** |  |  |  |
|  | 1 | Identify the study as an economic evaluation and specify the interventions being compared. | Yes |
| **Abstract** |  |  |  |
|  | 2 | Provide a structured summary that highlights context, key methods, results, and alternative analyses. | Yes |
| **Introduction** |  |  |  |
| **Background and objectives** | 3 | Give the context for the study, the study question, and its practical relevance for decision making in policy or practice. | Yes |
| **Methods** |  |  |  |
| **Health economic analysis plan** | 4 | Indicate whether a health economic analysis plan was developed and where available. | Yes |
| **Study population** | 5 | Describe characteristics of the study population (such as age range, demographics, socioeconomic, or clinical characteristics). | Yes |
| **Setting and location** | 6 | Provide relevant contextual information that may influence findings. | Yes |
| **Comparators** | 7 | Describe the interventions or strategies being compared and why chosen. | Yes |
| **Perspective** | 8 | State the perspective(s) adopted by the study and why chosen. | Yes |
| **Time horizon** | 9 | State the time horizon for the study and why appropriate. | Yes |
| **Discount rate** | 10 | Report the discount rate(s) and reason chosen. | Yes |
| **Selection of outcomes** | 11 | Describe what outcomes were used as the measure(s) of benefit(s) and harm(s). | Yes |
| **Measurement of outcomes** | 12 | Describe how outcomes used to capture benefit(s) and harm(s) were measured. | Yes |
| **Valuation of outcomes** | 13 | Describe the population and methods used to measure and value outcomes. | Yes |
| **Measurement and valuation of resources and costs** | 14 | Describe how costs were valued. | Yes |
| **Currency, price date, and conversion** | 15 | Report the dates of the estimated resource quantities and unit costs, plus the currency and year of conversion. | Yes |
| **Rationale and description of model** | 16 | If modelling is used, describe in detail and why used. Report if the model is publicly available and where it can be accessed. | Yes |
| **Analytics and assumptions** | 17 | Describe any methods for analysing or statistically transforming data, any extrapolation methods, and approaches for validating any model used. | Yes |
| **Characterising heterogeneity** | 18 | Describe any methods used for estimating how the results of the study vary for subgroups. | Yes |
| **Characterising distributional effects** | 19 | Describe how impacts are distributed across different individuals or adjustments made to reflect priority populations. | Yes |
| **Characterising uncertainty** | 20 | Describe methods to characterise any sources of uncertainty in the analysis. | Yes |
| **Approach to engagement with patients and others affected by the study** | 21 | Describe any approaches to engage patients or service recipients, the general public, communities, or stakeholders (such as clinicians or payers) in the design of the study. | Yes |
| **Results** |  |  |  |
| **Study parameters** | 22 | Report all analytic inputs (such as values, ranges, references) including uncertainty or distributional assumptions. | Yes |
| **Summary of main results** | 23 | Report the mean values for the main categories of costs and outcomes of interest and summarise them in the most appropriate overall measure. | Yes |
| **Effect of uncertainty** | 24 | Describe how uncertainty about analytic judgments, inputs, or projections affect findings. Report the effect of choice of discount rate and time horizon, if applicable. | Yes |
| **Effect of engagement with patients and others affected by the study** | 25 | Report on any difference patient/service recipient, general public, community, or stakeholder involvement made to the approach or findings of the study | Yes |
| **Discussion** |  |  |  |
| **Study findings, limitations, generalisability, and current knowledge** | 26 | Report key findings, limitations, ethical or equity considerations not captured, and how these could affect patients, policy, or practice. | Yes |
| **Other relevant information** |  |  |  |
| **Source of funding** | 27 | Describe how the study was funded and any role of the funder in the identification, design, conduct, and reporting of the analysis | Yes |
| **Conflicts of interest** | 28 | Report authors conflicts of interest according to journal or International Committee of Medical Journal Editors requirements. | Yes |

1. **Supplementary Table 2. Comparison of survival models.**

|  | **AIC** | | **BIC** | |
| --- | --- | --- | --- | --- |
|  | **DOS-CP** | **PLB-CP** | **DOS-CP** | **PLB-CP** |
| **OS-Overall Population** | | | | |
| Exponential | 688.76 | 968.53 | 691.55 | 975.52 |
| Weibull | 688.33 | 957.62 | 691.23 | 964.99 |
| Log-logistic | **686.25** | **953.22** | **688.73** | **960.19** |
| Lognormal | 686.19 | 654.63 | 689.42 | 961.41 |
| **OS-dMMR-MSI-H** | | | | |
| Exponential | 89.52 | 239.11 | 91.25 | 241.32 |
| Weibull | **84.68** | 236.62 | **85.19** | 237.46 |
| Log-logistic | 87.28 | **235.60** | 90.49 | **236.80** |
| Lognormal | 85.93 | 237.22 | 87.34 | 238.56 |
| **OS-pMMR-MSS** | | | | |
| Exponential | 595.22 | 721.66 | 602.36 | 729.58 |
| Weibull | **591.98** | 719.69 | **597.83** | 720.53 |
| Log-logistic | 593.07 | **718.71** | 598.46 | **719.06** |
| Lognormal | 594.19 | 719.91 | 599.89 | 724.52 |
| **PFS-Overall Population** | | | | |
| Exponential | 1075.42 | 1239.75 | 1080.31 | 1240.78 |
| Weibull | 1097.21 | 1263.56 | 1102.82 | 1270.76 |
| Log-logistic | **1070.71** | **1219.93** | **1076.66** | **1215.92** |
| Lognormal | 1071.35 | 1221.43 | 1078.47 | 1228.17 |
| **PFS-dMMR-MSI-H** | | | | |
| Exponential | 183.56 | 333.13 | 187.33 | 335.31 |
| Weibull | 182.4 | 331.66 | 184.25 | 335.08 |
| Log-logistic | **179.03** | **318.61** | **182.81** | **322.91** |
| Lognormal | 178.41 | 317.34 | 181.36 | 320.13 |
| **PFS-pMMR-MSS** | | | | |
| Exponential | 902.52 | 940.36 | 908.63 | 947.93 |
| Weibull | 904.93 | 944.89 | 911.15 | 951.44 |
| Log-logistic | **886.97** | **919.06** | **893.43** | **925.45** |
| Lognormal | 890.16 | 919.55 | 896.29 | 925.64 |

AIC, Akaike information criterion; BIC, Bayesian Information Criterion; dMMR-MSI-H, mismatch repair-deficient/microsatellite instability-high; DOS-CP, dostarlimab plus carboplatin-paclitaxel; PFS, progression-free survival; PLB-CP, placebo plus carboplatin-paclitaxel; pMMR-MSS, mismatch repair-proficient/microsatellite-stable; OS, overall survival.

**3.** **Supplementary Figure 1. Results of the survival curve fit the DOS-CP group and PLB-CP group.**

**
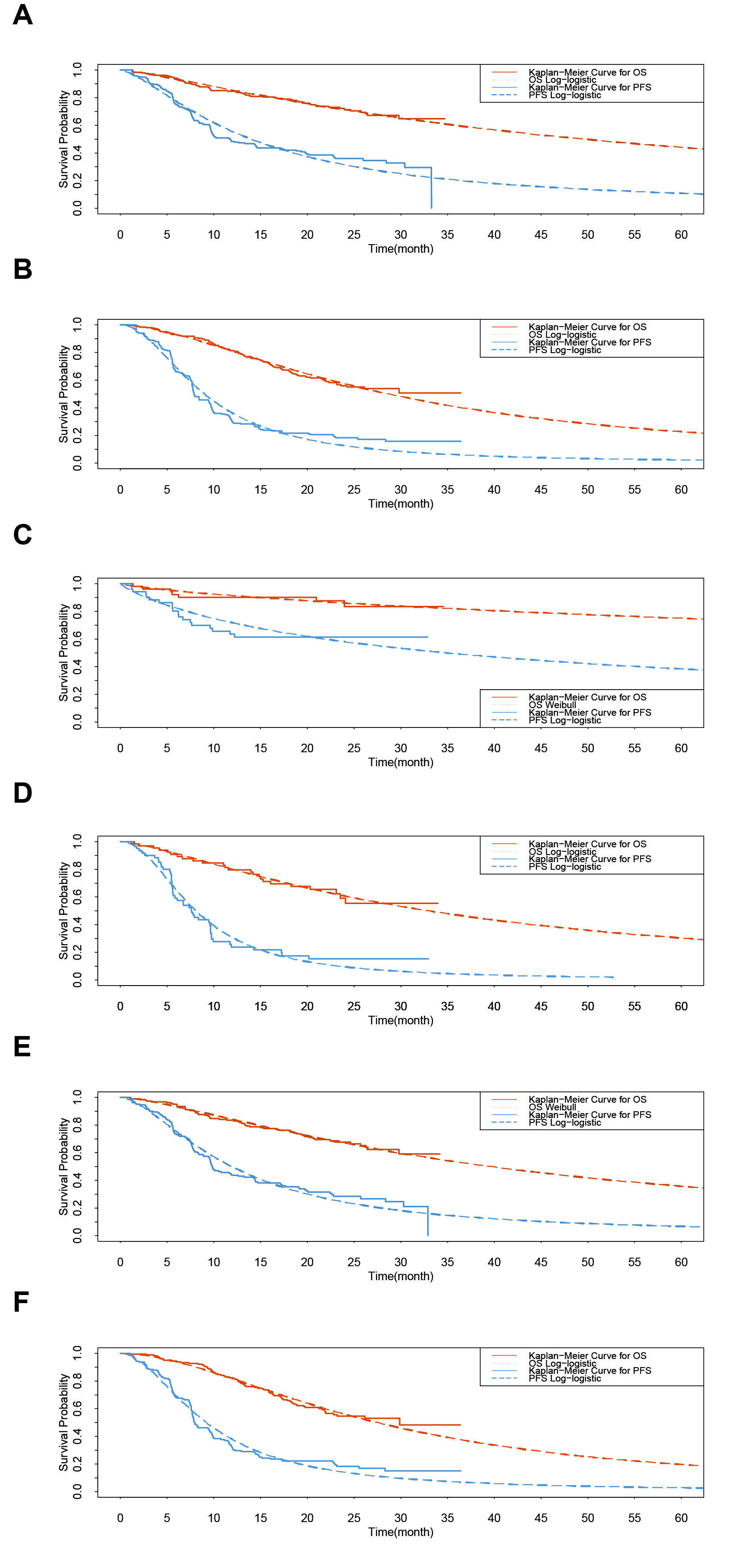
**

A, C, and E were survival curve fitting results in the DOS-CP group for patients with the overall population, dMMR-MSI-H subgroup, and pMMR-MSS subgroup, respectively. B, D, and F were survival curve fitting results in the PLB-CP group for patients with the overall population, dMMR-MSI-H subgroup, and pMMR-MSS subgroup, respectively. PFS, progression-free survival; OS, overall survival.

**4. Supplementary Figure 2: probabilistic sensitivity analysis for the overall population.**


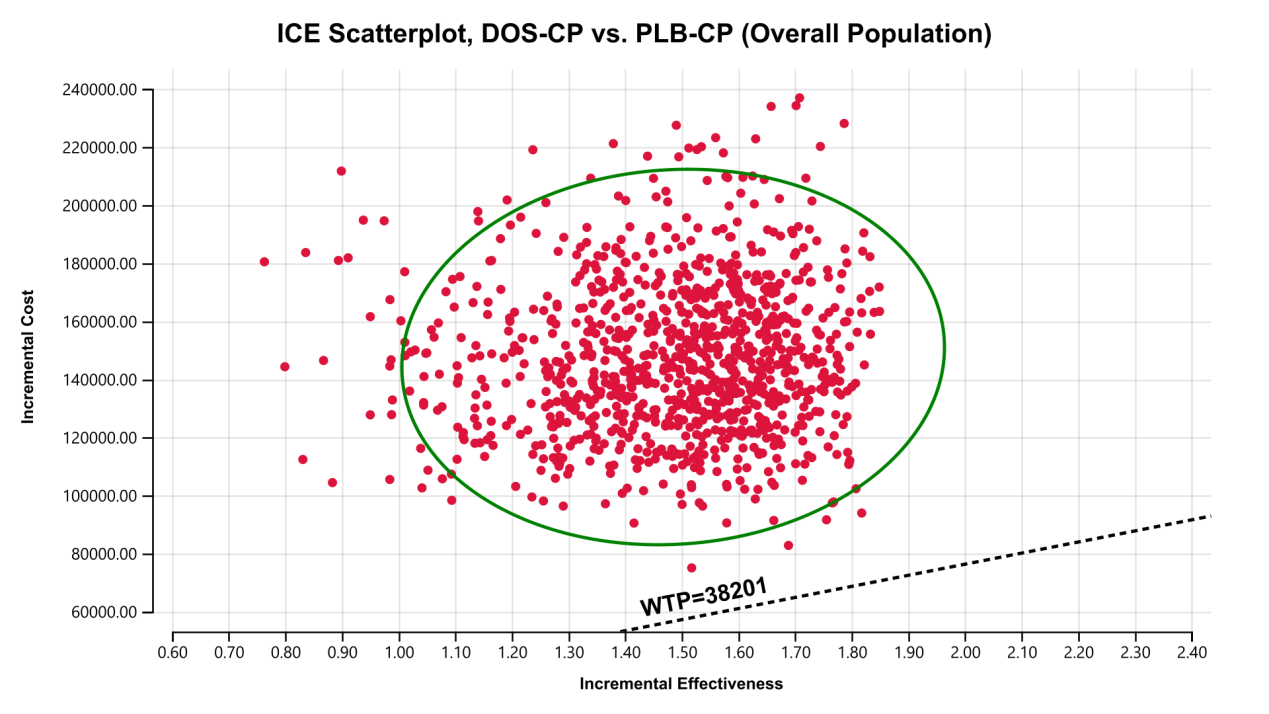


DOS-CP, dostarlimabplus carboplatin-paclitaxel; PLB-CP, placebo plus carboplatin-paclitaxel.

**5. Supplementary Figure 3: probabilistic sensitivity analysis for dMMR-MSI-H subgroup.**

**
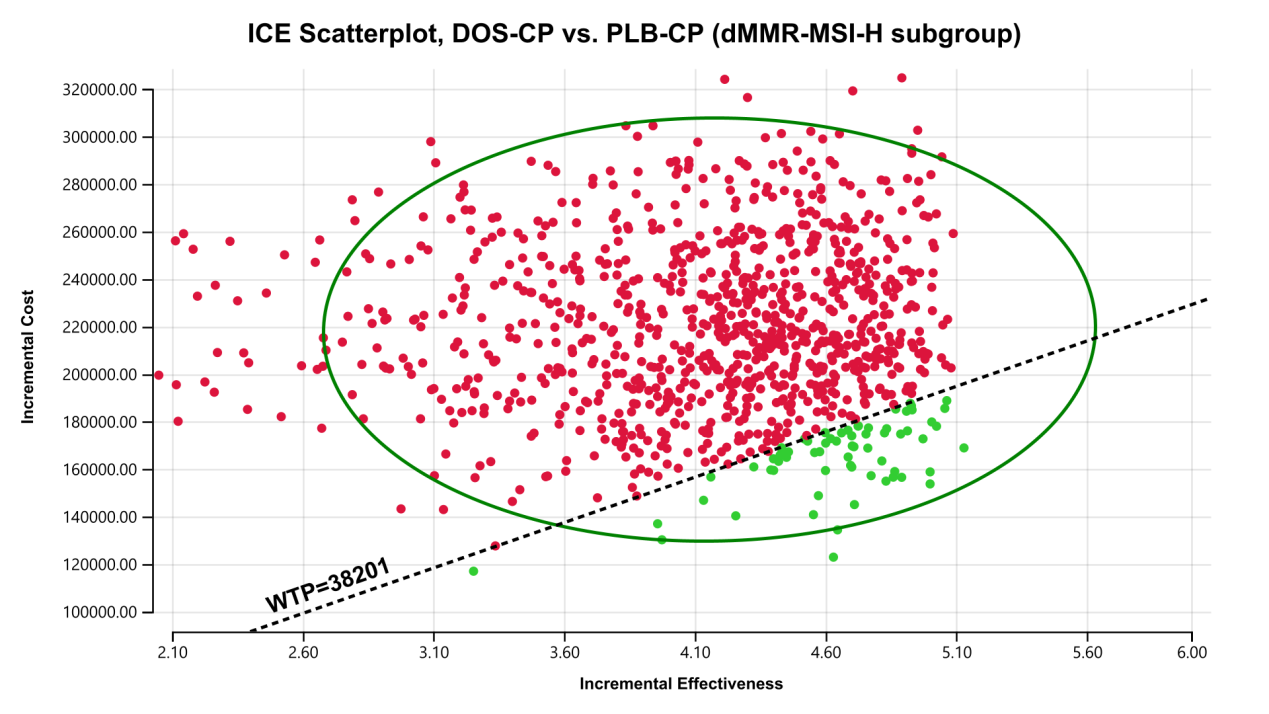
**

dMMR-MSI-H, mismatch repair-deficient/microsatellite instability-high; DOS-CP, dostarlimabplus carboplatin-paclitaxel; PLB-CP, placebo plus carboplatin-paclitaxel.

**6. Supplementary Figure 4: probabilistic sensitivity analysis for pMMR-MSS subgroup.**


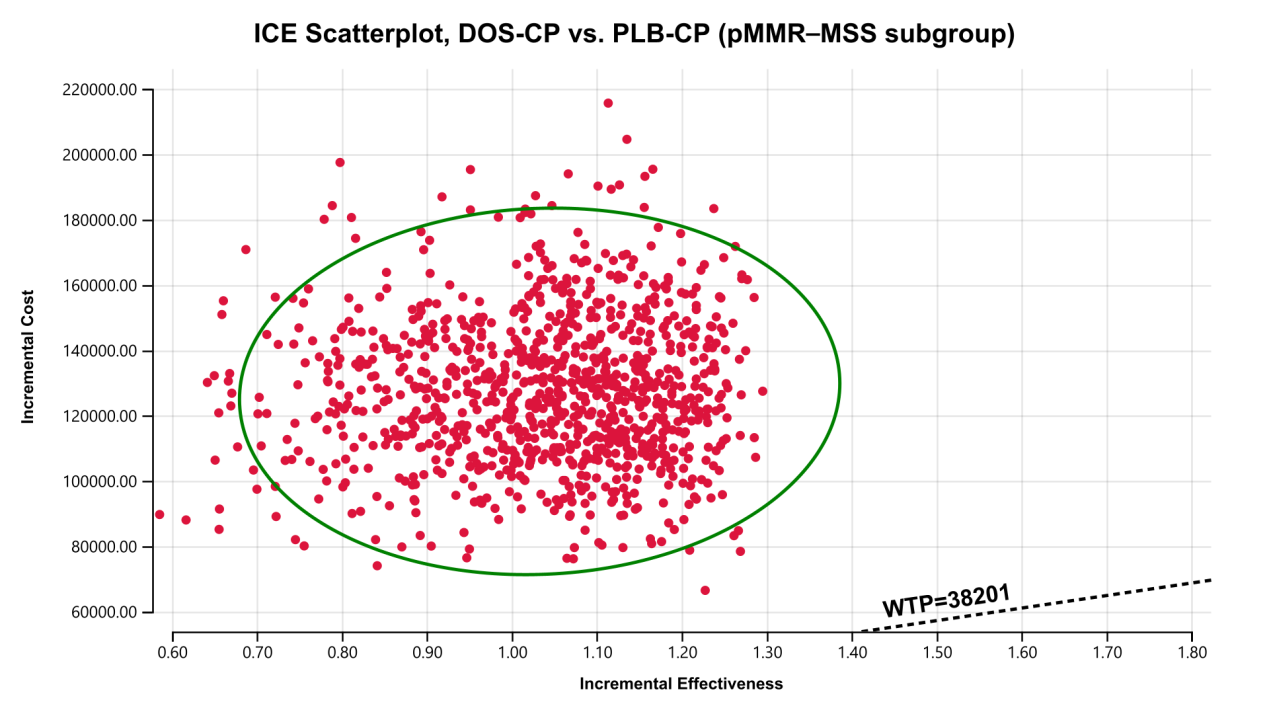


DOS-CP, dostarlimabplus carboplatin-paclitaxel; PLB-CP, placebo plus carboplatin-paclitaxel; pMMR-MSS, mismatch repair-proficient/microsatellite-stable.
